# Supplementary material for: Vertically stacked, low-voltage organic ternary logic circuits including nonvolatile floating-gate memory transistors
Source: Nat Commun. 2022 Apr 28;13:2305. doi: 10.1038/s41467-022-29756-w (PMC9051064; doi:10.1038/s41467-022-29756-w)
Supplement: Supplementary file 1 — Supplementary Information (pdf) [file 41467_2022_29756_MOESM1_ESM.pdf]

Supplementary Information

Vertically-Stacked, Low-Voltage Organic Ternary Logic Circuits Including Nonvolatile Floating-Gate Memory Transistors

Junhwan Choi,[1,†] *Changhyeon Lee,*[1,†] *Chungryeol Lee,*[1] *Hongkeun Park,*[1] *Seung Min Lee,*[1]
Chang-Hyun Kim,[2] *Hocheon Yoo,*[*,2] and *Sung Gap Im*[*,1,3]

[1] *Department of Chemical and Biomolecular Engineering*
Korea Advanced Institute of Science and Technology (KAIST)
291 Daehak-ro, Yuseong-gu, Daejeon 34141, Korea

[2] *Department of Electronic Engineering*
Gachon University
1342 Seongnam-daero, Seongnam, Gyeonggi-do, 13120, Korea

[3] *KAIST Institute For NanoCentury (KINC)*
Korea Advanced Institute of Science and Technology (KAIST)
291 Daehak-ro, Yuseong-gu, Daejeon, 34141, Korea

[†]These authors contributed equally to this work
Correspondence and requests for materials should be addressed to H.Y. (hyoo@gachon.ac.kr) or S.G.I. (sgim@kaist.ac.kr).

Schematic illustration of the device

The schematic illustration of the device is shown in Supplementary Fig. **1**. Note that the gate electrode of the flash memory and HTR was connected and the drain electrode of the flash memory and HTR was connected through the via-hole-less metal interconnection (Supplementary Fig. **1a**). Supplementary Fig. **1b** shows the optical microscopy image of the fabricated device and corresponding schematic illustration of the device according to the fabrication procedure.

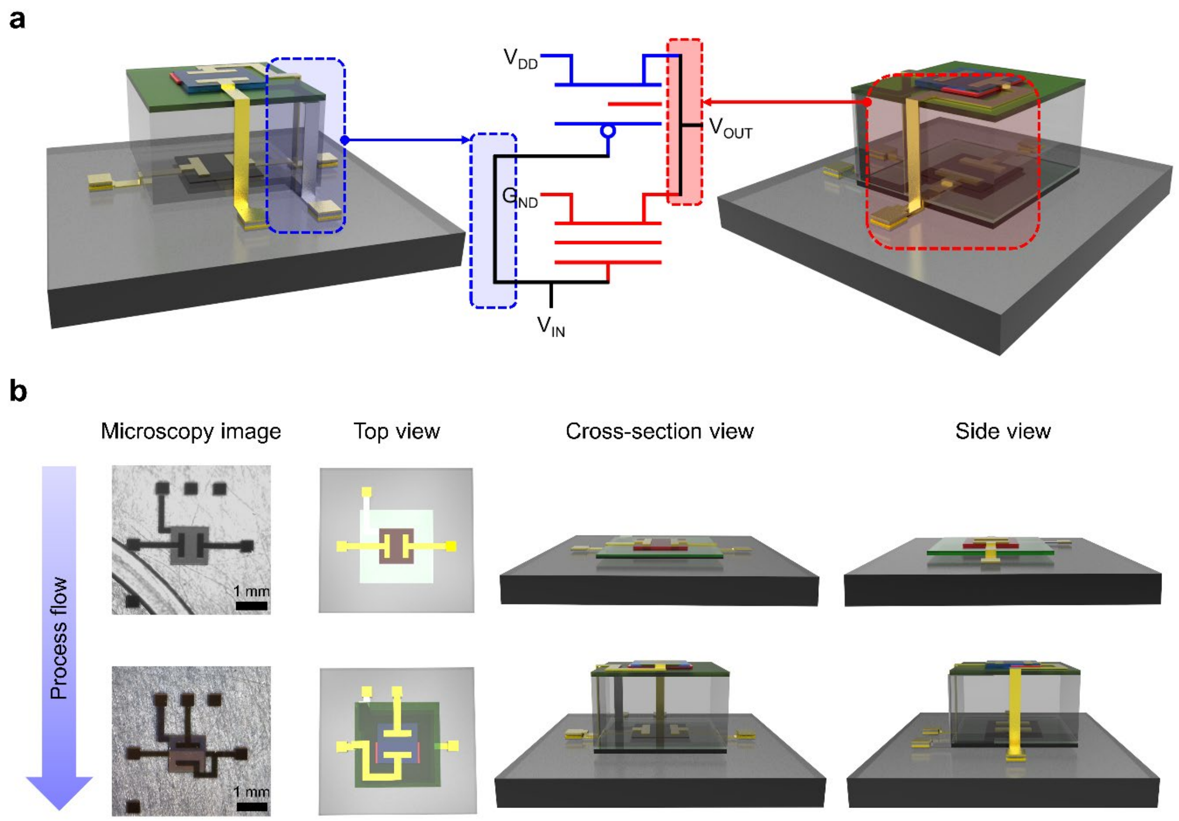

Supplementary Figure 1 | a. A schematic of the device and **b.** Microscopic image and schematic illustration of the device with different angles according to the fabrication procedure (top: organic flash memory in the 1st floor, bottom: organic HTR in the 2nd floor).

The chemical analysis of the polymer dielectric layers.

Supplementary Fig. **2** shows the chemical structures and X-ray photoelectron spectroscopy (XPS) analysis results of the polymer dielectric layers deposited via initiated chemical vapor deposition (iCVD) process, including poly(2-cyanoethyl acrylate-co-diethylene glycol divinyl ether) [p(CEA-co-DEGDVE)] with the optimum chemical composition (pC1D1)[1] and poly(1,3,5-trimethyl-1,3,5-trivinyl cyclotrisiloxane) (pV3D3).[2] The high-resolution C*1s* spectrum of pC1D1 blocking dielectric layer (BDL) was consistent with the previous report.[1] Also, the N*1s* peak was detected only in the XPS spectrum of pC1D1. In the XPS spectra of pV3D3 tunneling dielectric layer (TDL), interlayer dielectric (ILD) and gate dielectric layer (GDL) of the heterojunction transistor (HTR), C*1s* and Si*2p* peaks were commonly detected, whereas N*1s* peak was not observed.

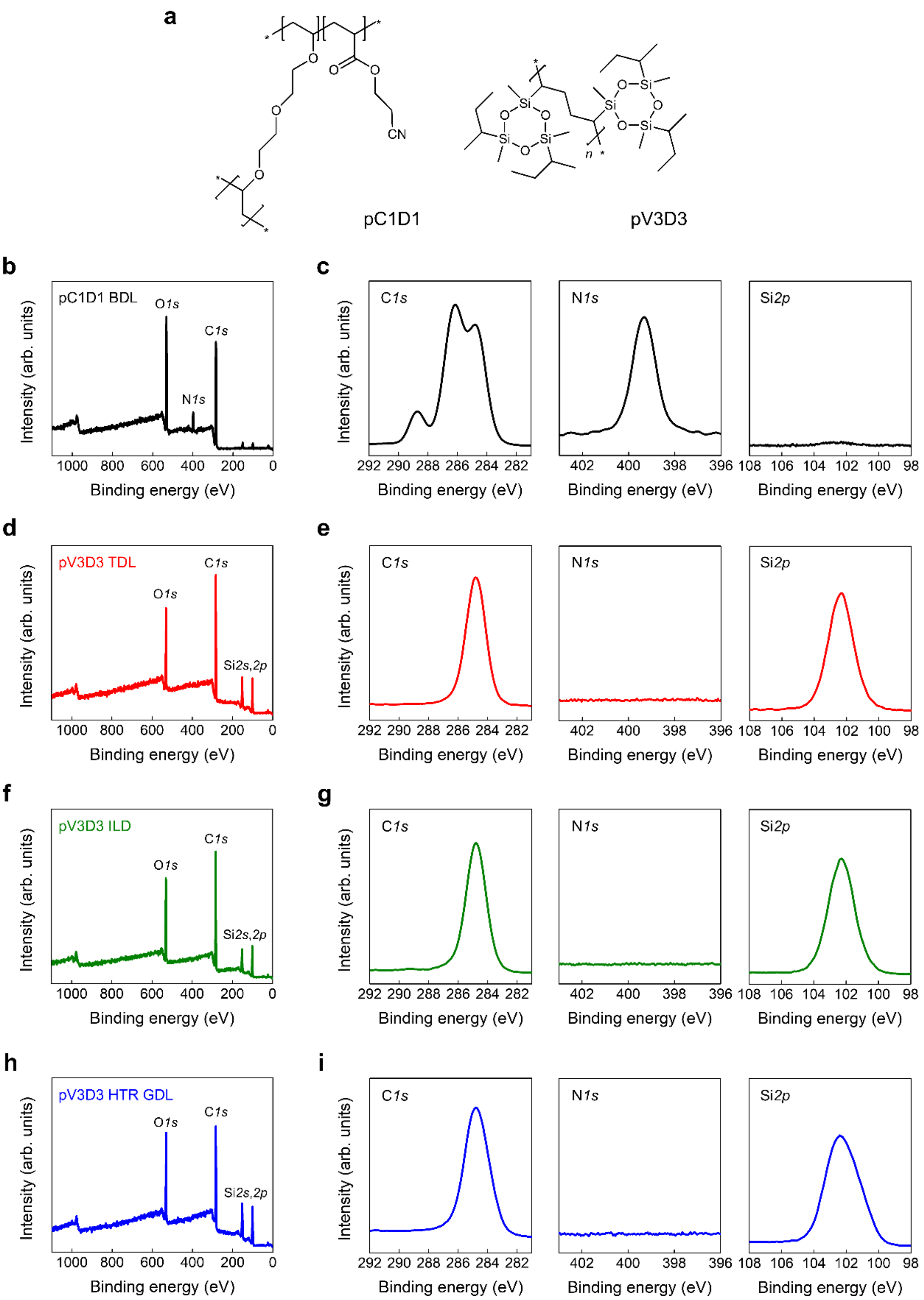

Supplementary Figure 2 | a. The chemical structures of polymer dielectric materials. **b**. The XPS survey scan and c, high-resolution C*1s*, N*1s* and Si*2p* spectra of the pC1D1 BDL. Those of **d-e**. pV3D3 TDL, **f-g**. pV3D3 ILD and **h-i**. pV3D3 GDL in the HTR.

The insulating performance of the dielectric layers.

The capacitance per unit area (C_i) with respect to frequency (f), electric field (E) and leakage current density (J) versus E of the dielectric layers are shown in Supplementary Fig. **3**. The pC1D1 BDL showed high C_i (54.1 nF/cm^2) even with 100 nm thickness (d) owing to its high dielectric constant (k = 6.2).[1] This high dielectric constant led high gate coupling ratio (α_{CR}), when combined with the relatively low dielectric constant of the pV3D3 TDL (k = 2.2).[3-4] The ultrathin (24 nm) pV3D3 TDL showed the Fowler-Nordheim (F-N)-like tunneling behavior in the E range from 3.5 to 6 MV/cm (Supplementary Fig. **3d**), which is fully consistent with our previous observation.[2] All the dielectric layers showed the excellent insulating performance (J < 10^{-7} A/cm^2 in the E range of ±4 MV/cm).

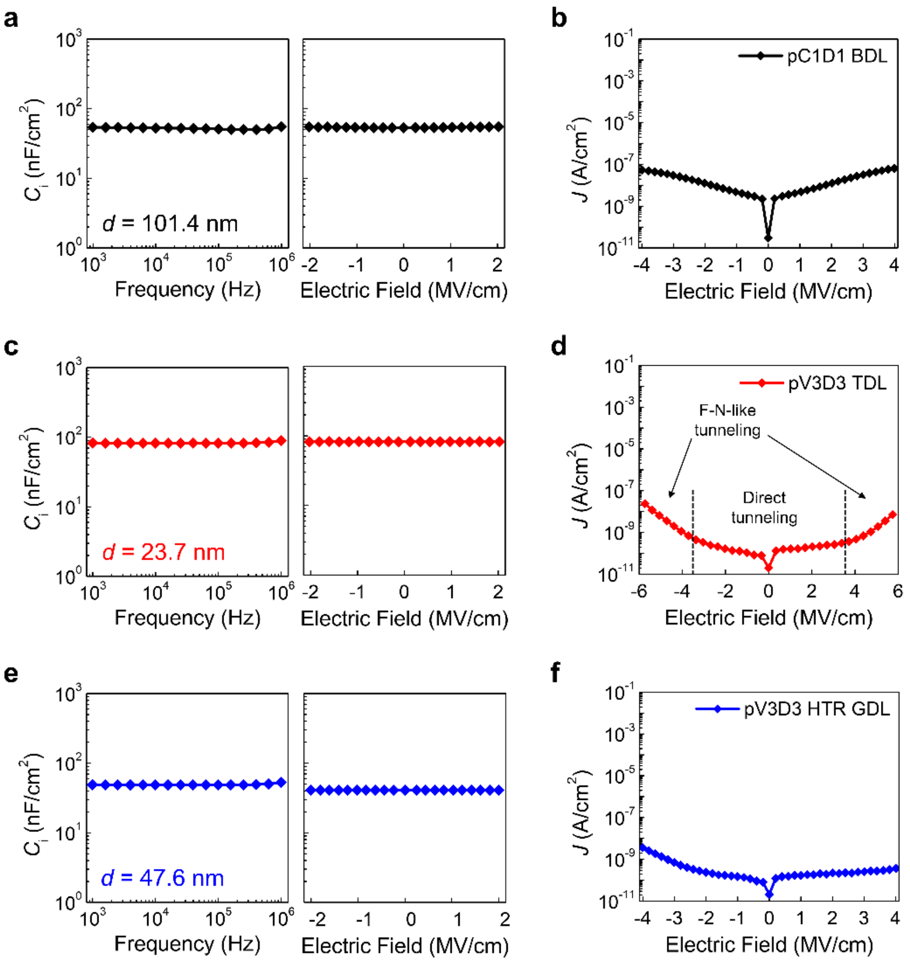

Supplementary Figure 3 | a. The C_i-f, C_i-E and **b.** J-E characteristics of the pC1D1 BDL (d ~101 nm), **c-d**. those of the pV3D3 TDL (d ~24 nm) and **e-f**. those of the pV3D3 GDL (d ~48 nm) in the HTR.

The high-resolution transmission electron microscope (HRTEM) analysis.

Supplementary Fig. **4a** shows the HRTEM images and energy dispersive spectroscopy (EDS) elemental mapping of the ternary logic inverter in three-dimensional structure (3D T-inverter). Supplementary Fig. **4b** and **4c** show the HRTEM images and EDS elemental mapping results of the flash memory in the first floor and the HTR in the second floor, respectively. The flash memory was fabricated based on *N*,*N*-ditridecylperylene-3,4,9,10-tetracarboxylic diimide (PTCDI-C13) and the HTR was fabricated based on PTCDI-C13 and dinaphtho[2,3-b:2′,3′-f]thieno[3,2-b]thiophene (DNTT). Both devices were well-isolated by 1 μm-thick ILD and all the layer showed no significant defect or intermixing in the EDS analysis.

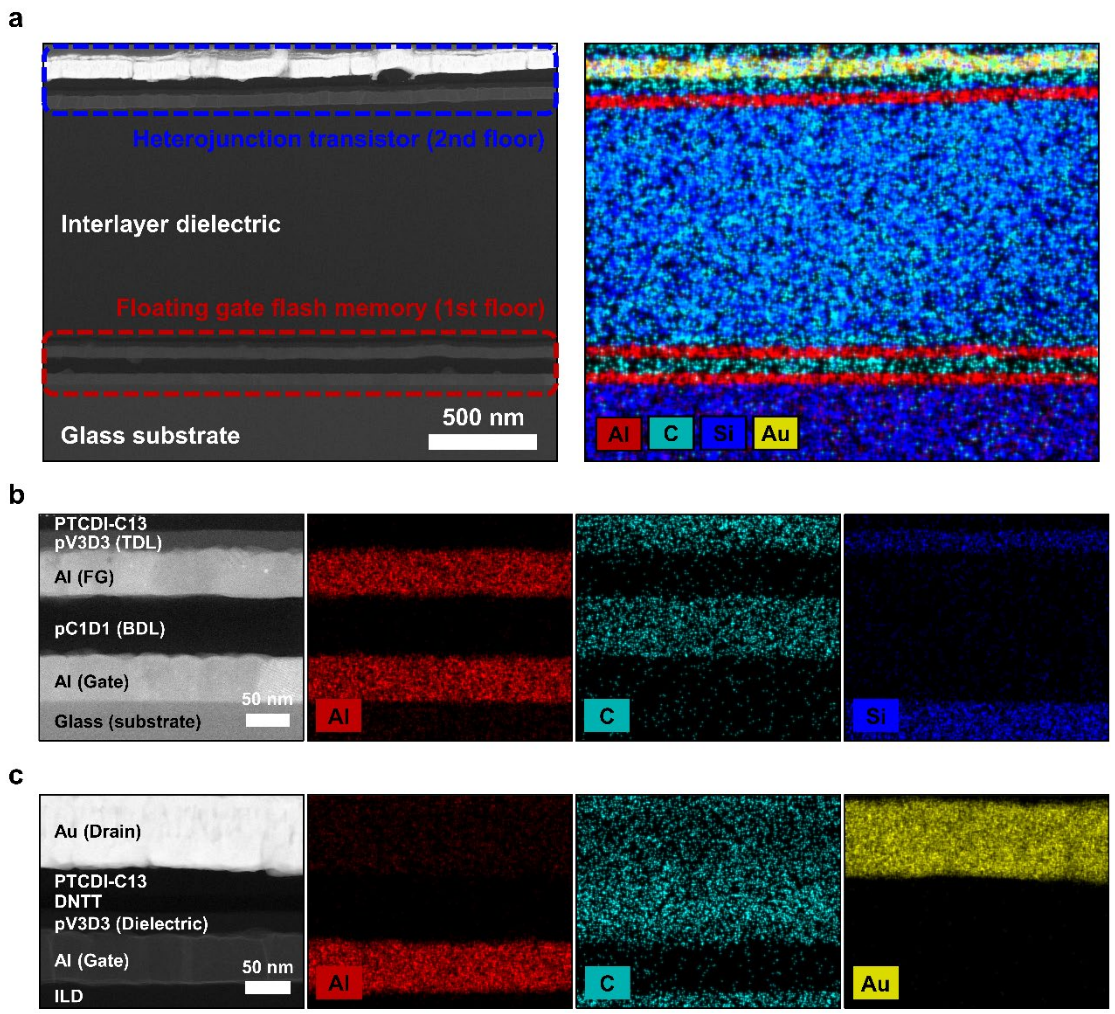

Supplementary Figure 4 | a. The HRTEM image and EDS elemental mapping result of the vertically stacked 3D T-inverter and those of the **b**. flash memory and **c**. HTR.

Thermal analysis of the flash memory under polymer ILD

To clarify the heat dissipation of the underlying floating-gate device, we performed a two-dimensional finite-element simulation to investigate the thermal properties of the proposed system.[5] The thermal boundary condition was set to 300 K at metal electrodes and the thermal conductivity and heat capacity of all organic materials (PTCDI-C13, pV3D3, pC1D1) was assumed to be 10^{-3} $Wcm^{-1}K^{-1}$ and 1 $Jcm^{-3}K^{-1}$, respectively.[6] Supplementary Fig. **5a**-**c** present the simulated physical quantities inside the PTCDI-C13 thin film at $V_G = V_D$ = 5 V. A strong accumulation of electrons at the PTCDI-C13/pV3D3(TDL) interface is clearly identifiable in Supplementary Fig. **5a** with an apparent channel pinch-off at the drain. This in turn resulted in pronounced Joule heating over the pinch-off region due to an elevated electric field, as shown in Supplementary Fig. **5b**. Supplementary Fig. **5c** shows the internal temperature distribution, where the direction and degree of heat dissipation from the thermal hot spot is distinctively visualized. Most importantly, despite the low thermal conductivity of organic materials and the use of a relatively thick interlayer dielectric polymer film on top of the device, the temperature rise was practically negligible – no larger than 0.1 K with the low operating voltage (5 V), as illustrated in Supplementary Fig. **5d**. Moreover, the temperature of the PTCDI-C13 flash memory fabricated with 1 μm-thick pV3D3 ILD was measured under the continuous operation at $V_G = V_D$=5 V. As shown in Supplementary Fig. **6**, the temperature change less than 0.1 K was observed throughout the whole continuous operation up to 5 hours, which is fully consistent with the result obtained by the two-dimensional finite-element simulation.

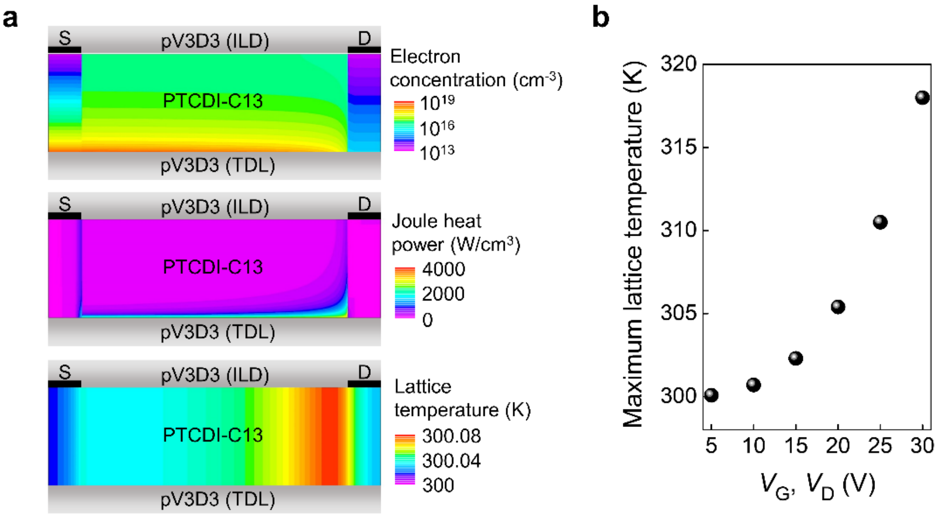

Supplementary Figure 5 | Simulated physical quantities inside a PTCDI-C13 transistor at V_G= V_D=5 V. **a**. electron concentration, **b**. Joule heat power, and **c**. lattice temperature. **d**. Dependence of the maximum lattice temperature on the driving voltages.

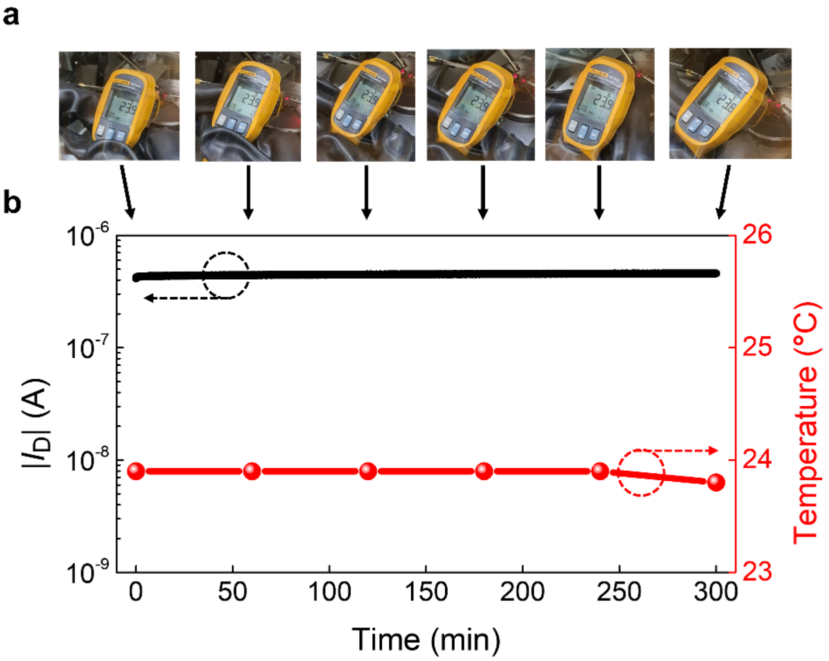

Supplementary Figure 6 | a. Photography for the measurement of the device temperature with an hour interval. **b.** The change in I_D and temperature of PTCDI-C13 flash memory with 1 μm-thick ILD during continuous operation at V_G= V_D=5 V.

The surface morphology analysis of the active layers and ILD.

Supplementary Fig. **7** exhibits the optical microscope (OM) images of the devices and atomic force microscope (AFM) images measured in the middle of active layer regions. PTCDI-C13 showed highly packed structure owing to the recrystallization process by post-thermal annealing (Supplementary Fig. **7a**). After the deposition of thick (~1 μm) ILD, the surface of the active layer region became flat with the root-mean-square roughness (R_q) of 1.77 nm (Supplementary Fig. **7b**). In the AFM image of the HTR at the middle of the heterojunction, two distinct regions with different DNTT grain sizes were clearly observed (Supplementary Fig. **7c**), due to the presence of the underlying partial PTCDI-C13 layer.[7]

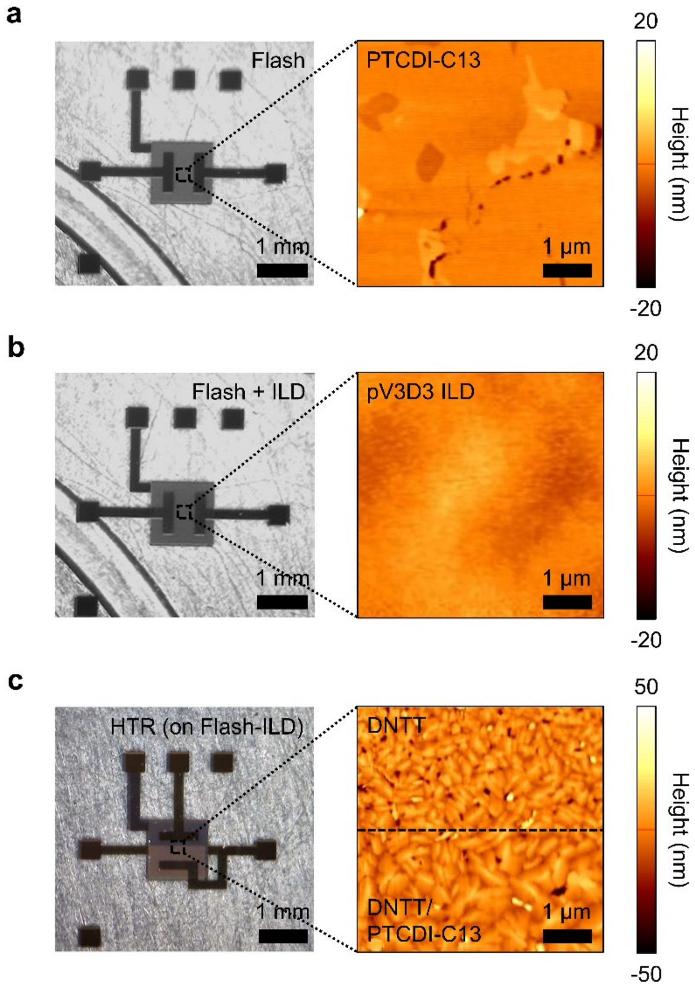

Supplementary Figure 7 | The OM images (left) and AFM images (right) at the middle of the active layer regions of **a**. the flash memory, **b**. ILD deposited on top of the flash memory and **c**, HTR.

The operating principle of the HTR

The HTR has a structure in which one electrode forms a contact with a stacked n-type/p-type semiconductor and the other electrode contacts a p-type semiconductor only. In the p–n junction at the channel, the band-to-band tunneling (BTBT) occurs as the conduction band in the intrinsic region aligns with the valence band in the p-type region.[8, 9] In the valence band of the p-type region, electrons tunnel into the conduction band of the intrinsic region and current can flow across the device.

The observed negative differential transconductance (NTC) implies the existence of the BTBT current. The operation principle can be divided into three different regions, as shown in Supplementary Fig. **8**:

i) 0 V > V_G > −1.5 V: Electron BTBT current occurs, and hole carriers begin to accumulate. Both the BTBT current and p-channel current contribute to I_D, exhibiting a peak current (3.5×10^{-8} A at V_G = −1.5 V).

ii) −1.5 V > V_G > −2.8 V: The BTBT current decreased as the n-type PTCDI-C13 is depleted by the negative gate voltage bias, resulting in the decrease of I_D with a valley current (2×10^{-9} A at V_G = −2.8 V).

iii) V_G < −2.8 V: The n-type PTCDI-C13 is completely depleted, and only the p-channel current contributes to I_D.

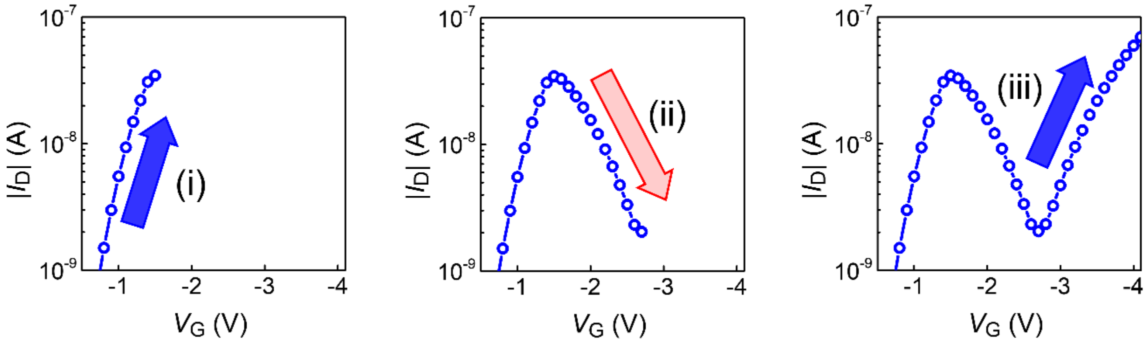

Supplementary Figure 8 | The operating principle of the HTR divided by three distinguishable regimes.

The uniformity analysis of HTR.

Supplementary Fig. **9a** shows the threshold voltage (V_T) and on/off current ratio (I_{on}/I_{off}) distribution of the fabricated 11 HTR devices. High degree of uniformity in V_T (-0.81±0.09 V) was obtained, however, relatively large I_{on}/I_{off} variation was observed among the HTR devices, because even slight variation in valley voltage (V_{valley}) can lead a large difference of I_{on}. Nevertheless, all the devices showed I_{on}/I_{off} higher than 10^4, indicating clear switching characteristics. As shown in Supplementary Fig. **9b**, the HTR devices exhibited the excellent device-to-device uniformity in peak voltage (V_{peak}) (-1.46±0.10 V), V_{valley} (-2.55±0.11 V) and resulting negative transconductance (NTC) length (1.09±0.10 V).

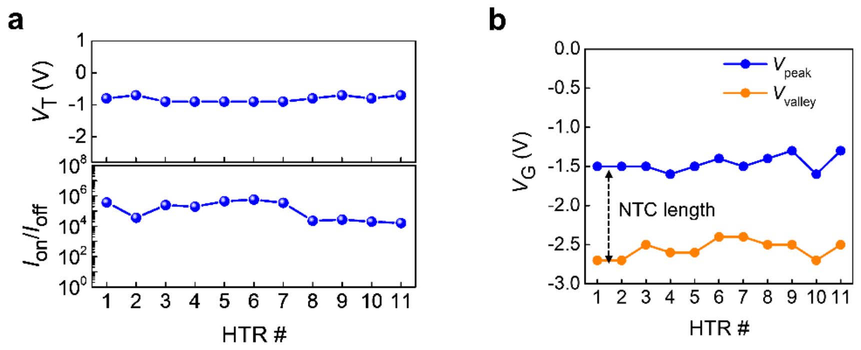

Supplementary Figure 9 | a. The V_T (top), I_{on}/I_{off} (bottom) and **b.** V_{peak} and V_{valley} variation of the 11 HTR devices.

The electrical characteristics of the transistors and inverter with different measuring speed.

The transfer characteristics of the flash memory and HTR measured with each gate voltage (V_G) sweeping speed were shown in Supplementary Fig. **10**. The VTCs of the T-inverter with each input voltage (V_{IN}) sweeping speed were also shown in Supplementary Fig. **11**. No notable hysteresis was observed, and extremely low gate leakage current was fully maintained regardless of the sweeping speed in the transfer curves of the transistors. The T-inverter also showed only negligible amount of hysteresis with the sweeping speed as low as 0.06 V/s.

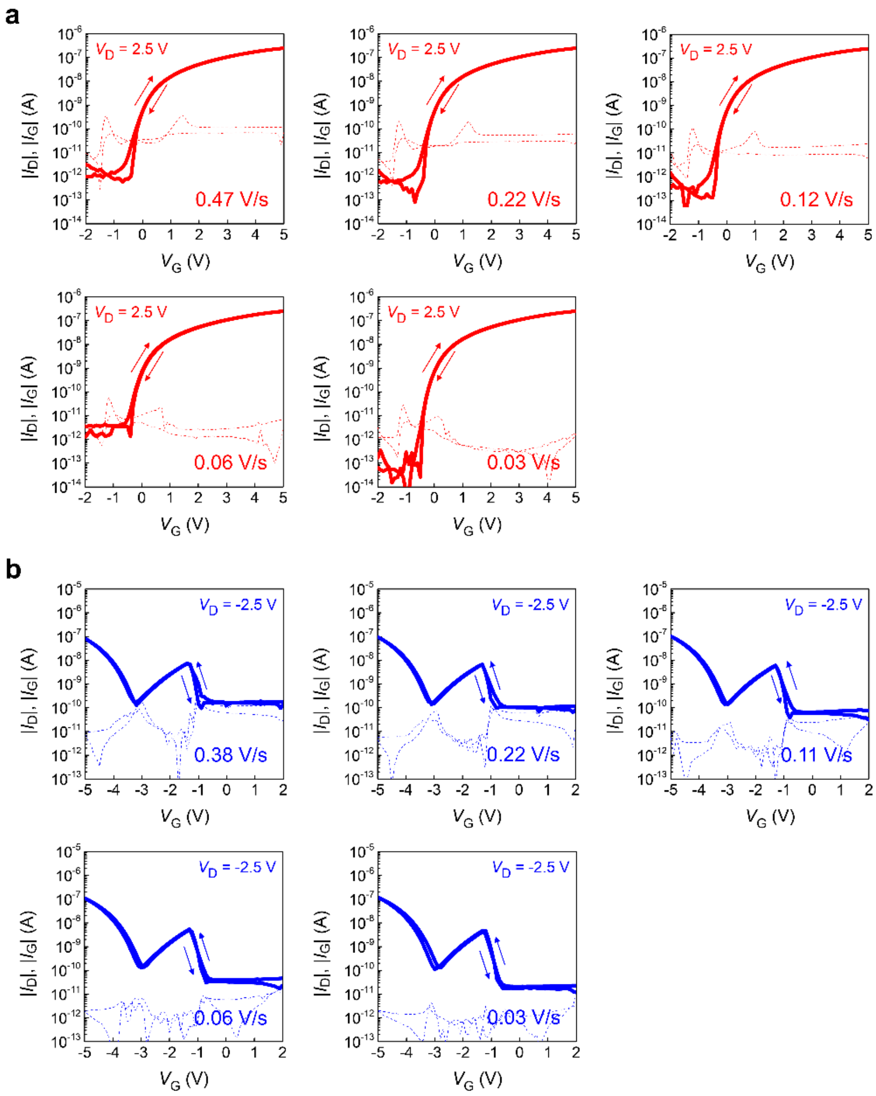

Supplementary Figure 10 | The transfer curves of the **a.** flash memory and **b.** HTR with different sweeping speed.

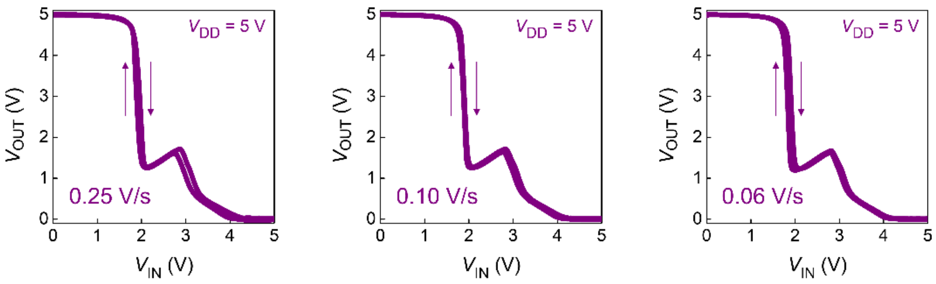

Supplementary Figure 11 | The VTCs of the T-inverter with different sweeping speed.

Band structure of the flash memory

A schematic electronic energy band diagrams of the flash memory of the initial state and programming/erasing operations are shown in Supplementary Fig. **12**. Charges accumulated in the active layer can be injected into floating-gate (FG) in programming operation or the stored charges can be removed from FG in erasing operation (Supplementary Fig. **12c-d**). Those operations were based on the F-N-like tunneling through TDL, which could be achieved with low programming/erasing voltage (V_{prg}/ V_{ers}) resulting from the high α_{CR}.[3-4]

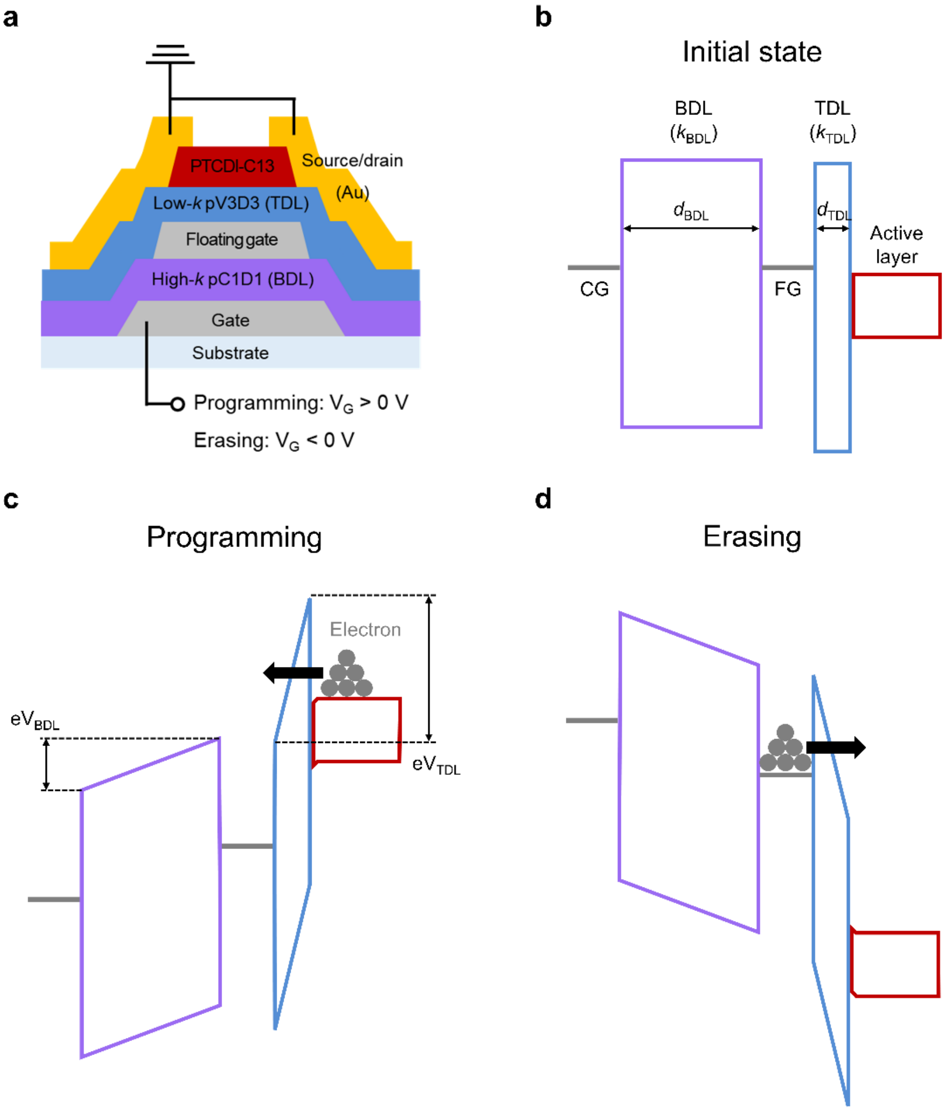

Supplementary Figure 12 | a. A schematic illustration of the flash memory operation and corresponding band diagram **b.** with initial state, **c.** programming operation and **d.** erasing operation.

The analysis on switching speed of the flash memory

The switching speed of the flash memory device was analyzed by applying V_{prg}/V_{ers} with different time scale (Supplementary Fig. **13a** and **b**). The V_{prg} and V_{ers} was optimized to +17 and -9 V, respectively, to achieve sufficient on/off current ratio. The V_T shift (ΔV_T) started to occur at the pulse width of 1 ms and the ΔV_T was systematically increased with the increasing time (Supplementary Fig. **13c**). With the pulse width of 10 s, ΔV_T was as high as 3.67 and -2.44 V in programming and erasing operation, respectively.

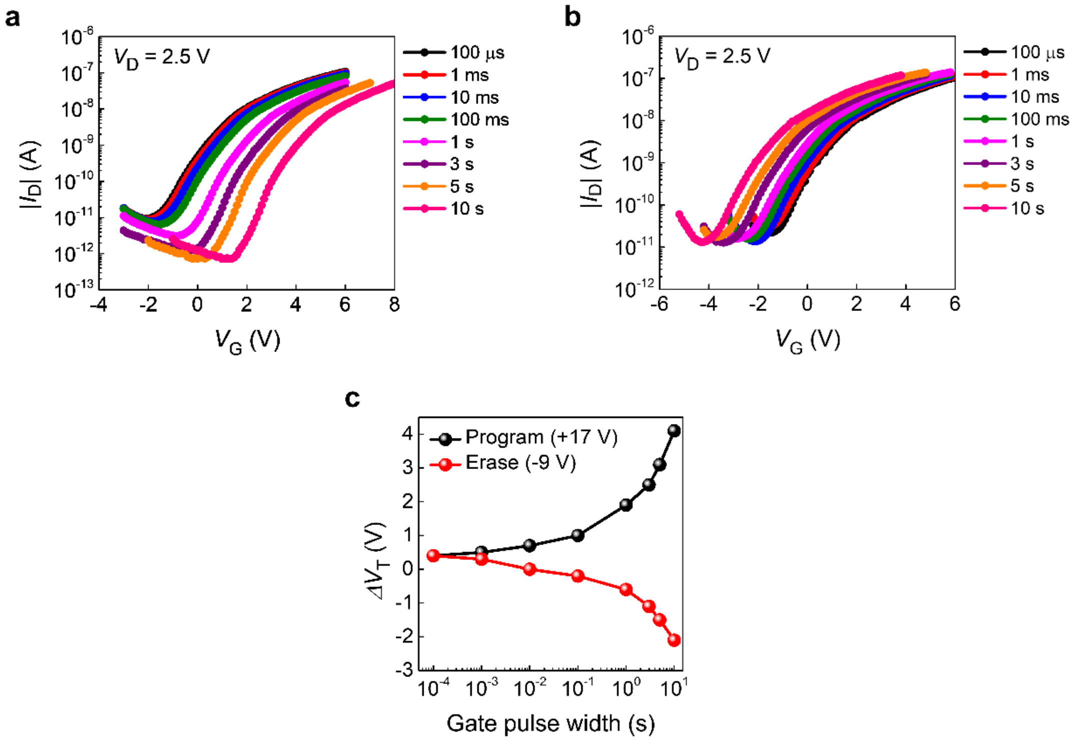

Supplementary Figure 13 | The transfer curves in a. programming and **b.** erasing operation. **c**, The ΔV_T with the applied V_{prg}/V_{ers} with different gate pulse width.

The analysis on cyclic endurance of the flash memory

The cyclic endurance of the flash memory was analyzed by repeatedly applying V_{prg}/ V_{ers} (Supplementary Fig. **14a**). The device showed excellent cyclic stability with the clearly distinguishable I_{on} and I_{off} variation over 50 cycles (Supplementary Fig. **14b**), mainly resulting from the outstanding insulating performance of the iCVD dielectrics.

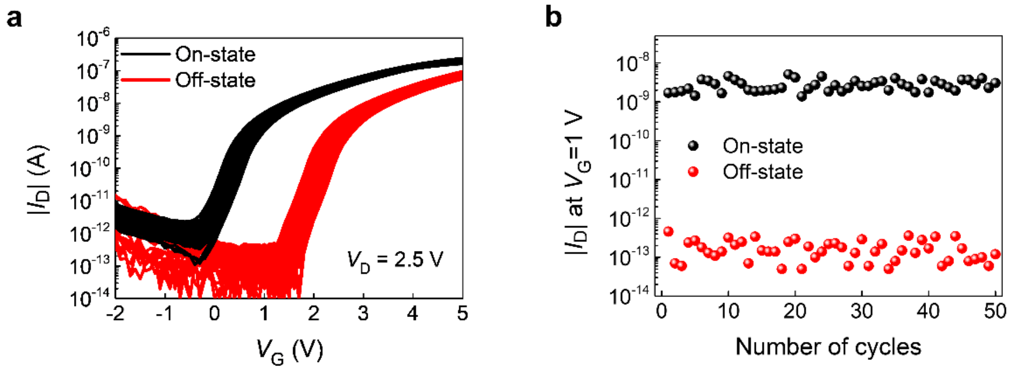

Supplementary Figure 14 | a. The transfer curves of repetitive programming and erasing operations up to 50 cycles. **b.** The change in I_D at V_G=1 V according to the number of endurance cycles.

Output characteristics of the flash memory and HTR

The output characteristics of the HTR and flash memory with each programming state is shown in Supplementary Fig. **15**. In the flash memory, I_D decreased significantly after applying V_{prg} of +19 V compared to that observed in the pristine flash memory, which supports that the channel conductance was controlled successfully by the programming operation of the flash memory.

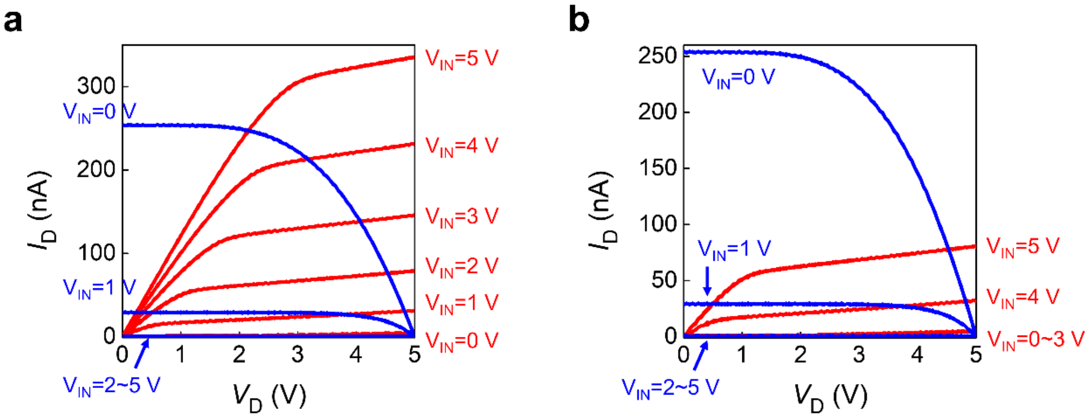

Supplementary Figure 15 | The output curves of the HTR (blue) and flash memory (red) in **a.** pristine and **b.** the programmed state (V_{prg}=+19 V).

The transfer characteristics of the HTR with different V_D

To investigate the origin that causes the shift of the intermediate logic state, we measured the transfer characteristics of the HTR with different V_D, because V_D of the HTR is determined by the difference between the supply voltage and the intermediate logic value (Supplementary Fig. **16a**). With the decreasing $|V_D|$ of the HTR, the NTC region was shifted toward lower $|V_G|$, which led the shift of the intermediate logic state to higher V_{IN}. Nevertheless, the length of the NTC region was fully preserved (Supplementary Fig. **16b**) The shift of the NTC region is most likely resulting from the reduced electron injection with the decreasing gate-to-drain voltage (V_{GD}) so that the depletion of n-type PTCDI-C13 started to occur at lower $|V_G|$.

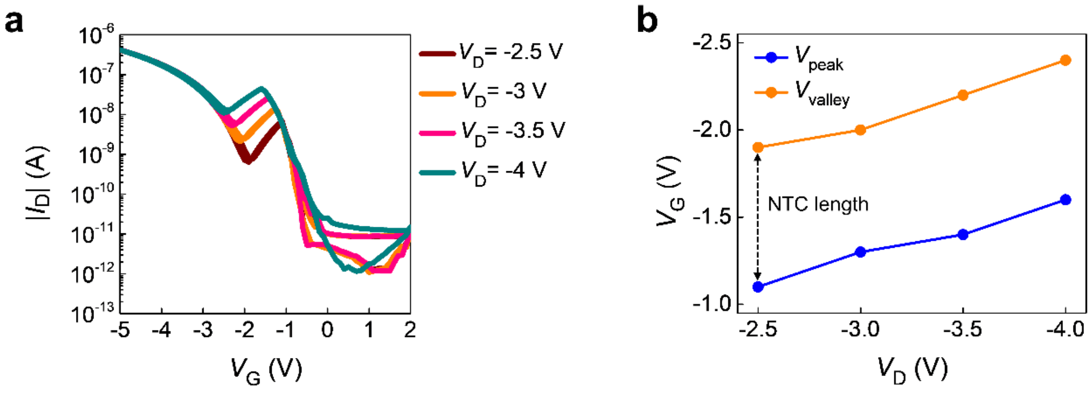

Supplementary Figure 16 | a. The transfer characteristics of the HTR with different V_D. **b**. the change in V_{peak} and V_{valley} with respect to V_D.

The overlapped transfer curves of the transistors and resulting VTC of the T-inverter according to memory programming state

Supplementary Fig. **17a-d** shows the overlapped transfer curves of the flash memory and HTR to analyze the intermediate logic state according to the memory programming state. The VTCs of the T-inverter was also shown in Supplementary Fig. **17e-h** along with each memory programming state. With the low V_{prg} (+14 and +16 V), the V_G region where the flash memory and HTR exhibited similar I_D (less than an order of magnitude difference) was relatively wide (~1.2 V) (Supplementary Fig. **17a, b**). In those V_G regions, the flash memory showed slightly higher I_D compared to that of the HTR, resulting in low intermediate logic value (1.09 and 1.47 V at V_{prg} = +14 and +16 V, respectively). However, with the V_{prg} of +19 V, both transistors showed quite similar I_D values in the NTC of the HTR, which produced the ideal V_{OUT} value (~ V_{DD}/2) in the intermediate logic state. In this optimum programming state, the V_{IN} range of the intermediate logic state decreased slightly to ~0.9 V, because only the NTC region in the HTR can represent the intermediate logic state.

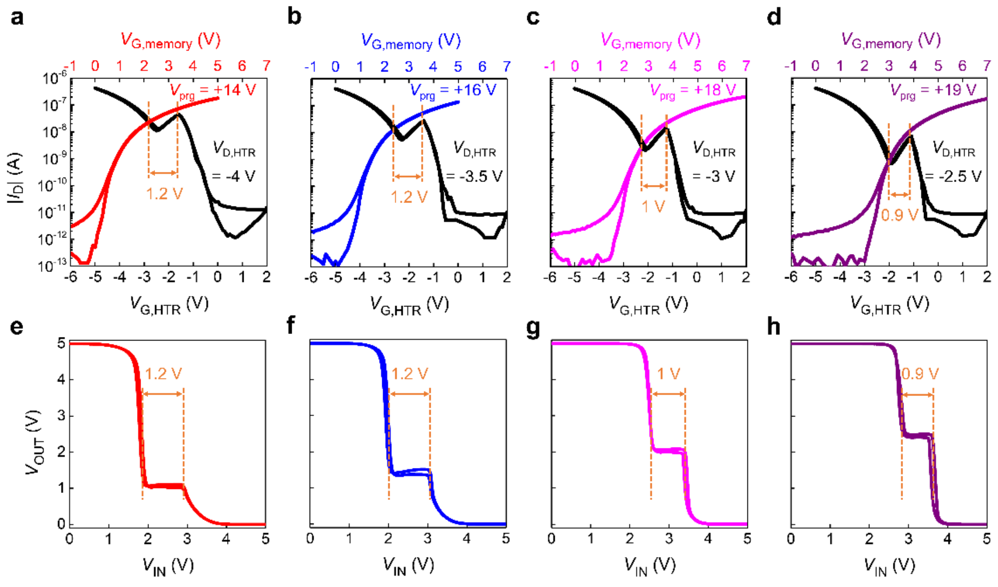

Supplementary Figure 17 | The overlapped transfer characteristics of the flash memory according to V_{prg} and the HTR with different V_D; **a**. V_{prg}=+14 V, $V_{D,HTR}$=-4 V, **b**. V_{prg}=+16 V, $V_{D,HTR}$=-3.5 V, **c**. V_{prg}=+18 V, $V_{D,HTR}$=-3 V and **d**. V_{prg}=+19 V, $V_{D,HTR}$=-2.5 V. **e-h**. The corresponding VTC of the T-inverter.

The electrical characteristics of the 3D T-inverter with flash memory programming

Supplementary Fig. **18** shows the change in voltage transfer characteristics (VTCs) and DC gain profiles of the 3D T-inverter according to V_{prg} of the flash memory. The intermediate logic value increased gradually with the increasing V_{prg} in incremental step pulse programming (ISPP) of the flash memory. The maximum gain value in the first peak (1st gain) value decreased and that in the second peak (2nd gain) value was increased with the increasing V_{prg}, due to the increased intermediate logic value.

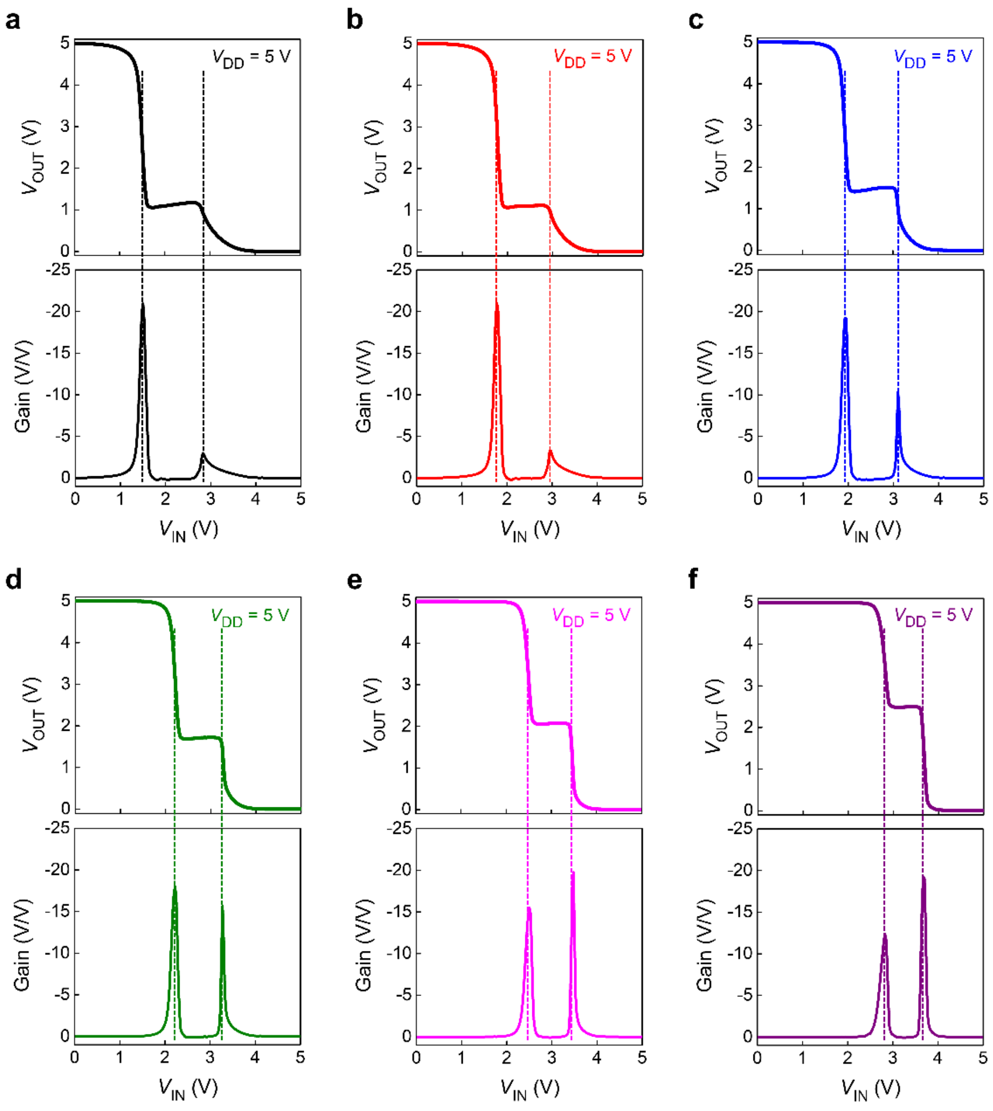

Supplementary Figure 18 | The VTC (top) and gain profile (bottom) of the 3D T-inverter according to the flash memory programming states: **a**. pristine and after programming with V_{prg} of **b**. +14 V, **c**. +16 V, **d**. +17 V, **e**. +18 V and **f**. +19 V.

The electrical characteristics of the flash memory and 3D T-inverter in erasing operation

The change in electrical characteristics of the flash memory in erasing operation was also investigated via incremental step pulse erasing (ISPE) (Supplementary Fig. **19a**). The transfer curves shifted gradually toward negative direction with the increasing $|V_{ers}|$ (Supplementary Fig. **19b**). Along with the change in flash memory, the VTC of the T-inverter also shifted systematically, although the intermediate logic value decreased with the increasing $|V_{ers}|$ and even full-swing operation was not retained with V_{ers} of -8 V (Supplementary Fig. **19c** and **d**). The 2nd gain decreased gradually with the increasing $|V_{ers}|$ (Supplementary Fig. **19e**). There was no notable change in the 1st gain according to V_{ers} except for V_{ers} of -8 V, because the V_{OUT} value at V_{IN}=0 V was reduced with higher $|V_{ers}|$, majorly due to far higher channel conductance of the flash memory than that of the HTR. The V_{OUT} value at V_{IN}=3.25 V also decreased with the memory erasing state, compared to that in the pristine 3D T-inverter in transient operation (Supplementary Fig. **19f**).

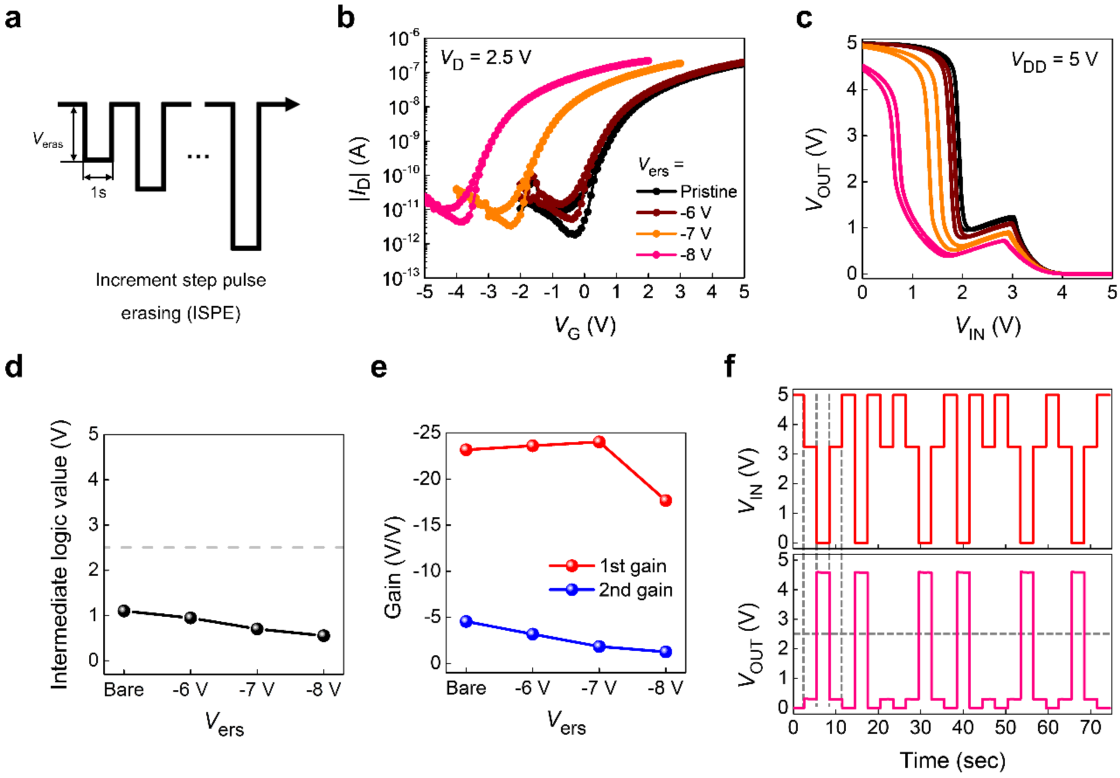

Supplementary Figure 19 | a. A schematic of ISPE operation. **b**. The transfer characteristics of flash memory and **c**. VTCs of the T-inverter, **d**. intermediate logic value and **e**. 1st and 2nd gain values according to V_{ers}. **f**. Transient measurement results of the T-inverter in the memory erasing state with the V_{ers} of -8 V.

The electrical characteristics of the 3D T-inverter according to flash memory erasing

Supplementary Fig. **20** shows the change in VTCs and DC gain profiles of the 3D T-inverter according to the V_{ers} of the flash memory via ISPE. The 1st gain value increased slightly with the increasing $|V_{ers}|$ except for V_{ers} of -8 V due to the reduced V_{OUT} value at V_{IN} = 0 V. The 2nd gain value decreased with the increasing $|V_{ers}|$.

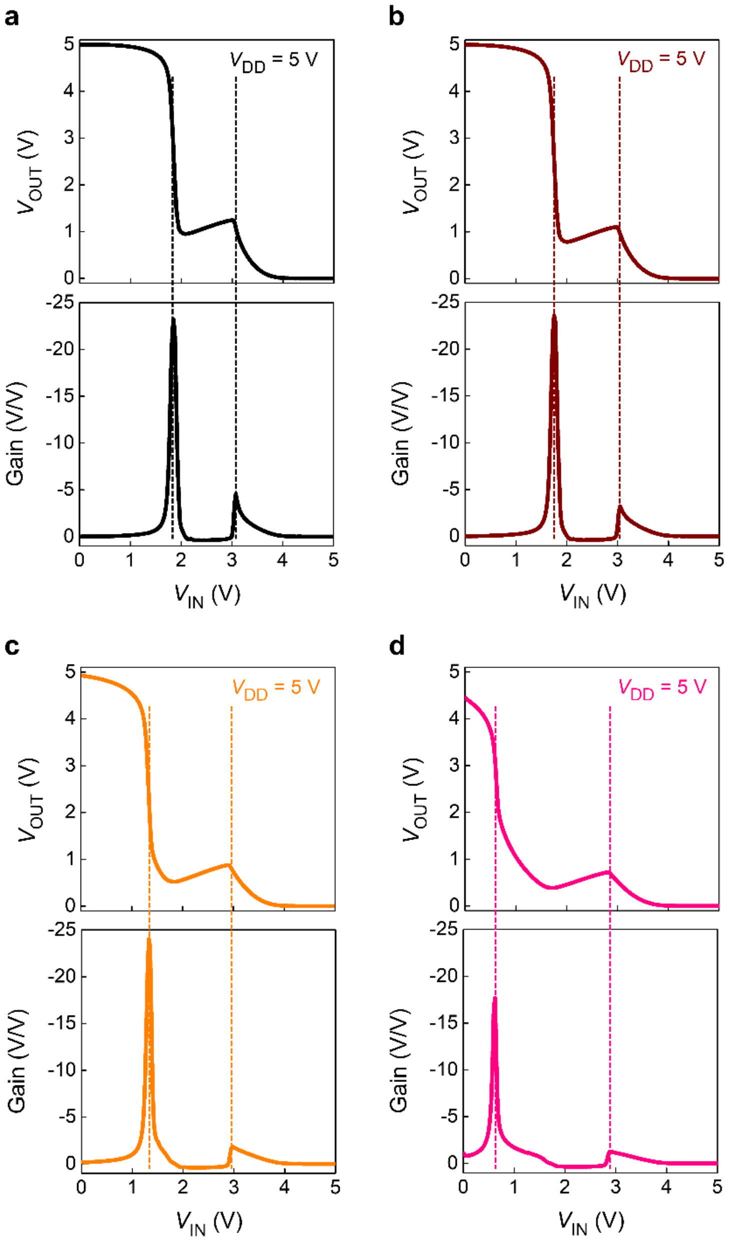

Supplementary Figure 20 | The VTC (top) and DC gain profile (bottom) of the 3DT-inverter according to the flash memory erasing states: **a**. pristine and after erasing with V_{ers} of **b**. -6 V, **c**. -7 V, **d**. -8 V.

The analysis on the switching speed of the T-inverter

To investigate the switching speed of the 3D T-inverter with the optimum programming state, we measured the V_{OUT} in the response to the applied V_{IN} according to time (Supplementary Fig. **21**). The swiching speed of the T-inverter was calcuated as the difference between the time when a 10% increase in V_{OUT} occurs and the time when a 90% of the terminal V_{OUT} was achieved.

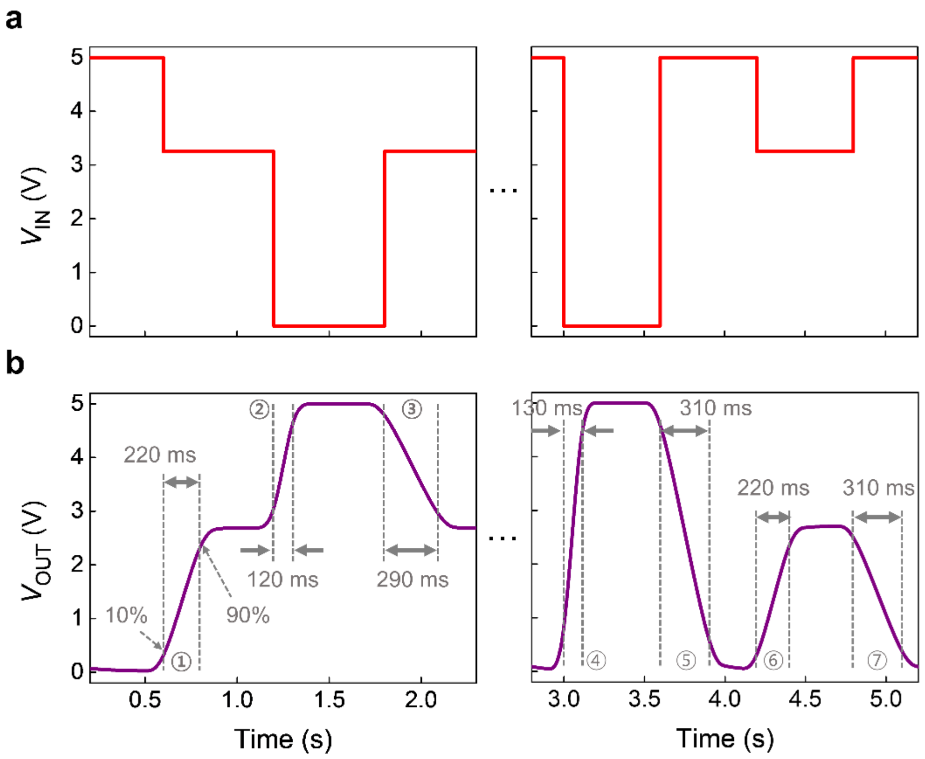

Supplementary Figure 21 | a. The applied V_{IN} and **b.** V_{OUT} in response to the applied V_{IN} of the T-inverter with the optimum programming state.

The retention characteristics of the flash memory in erasing operation

The retention characteristics in memory erasing operation were also investigated. The change in transfer characteristics with time after applying the maximum V_{ers} (-8 V) are shown in Supplementary Fig. **22a**, which showed negligible change over time with only 0.6 V of ΔV_T after 10^5 s. This excellent retention performance led little change in the electrical characteristics of the 3D T-inverter over time (Supplementary Fig. **22b-c**). Also, there was no notable device degradation such as hysteresis behavior. The change in intermediate logic value was less than 0.10 V even after 10^5 s (Supplementary Fig. **22d**). Even with the extremely small change in the transfer curves, V_{OUT} at V_{IN}=0 V was recovered from 4.45 to 4.78 V in the VTC after 10^5 s, which induced the enhanced voltage swing and the improved 1st gain value from -17.7 to -23.1 V/V (Supplementary Fig. **22e**). Nevertheless, there was still negligible change in the 2nd gain value (less than 0.5 V/V) throughout the whole measurement time.

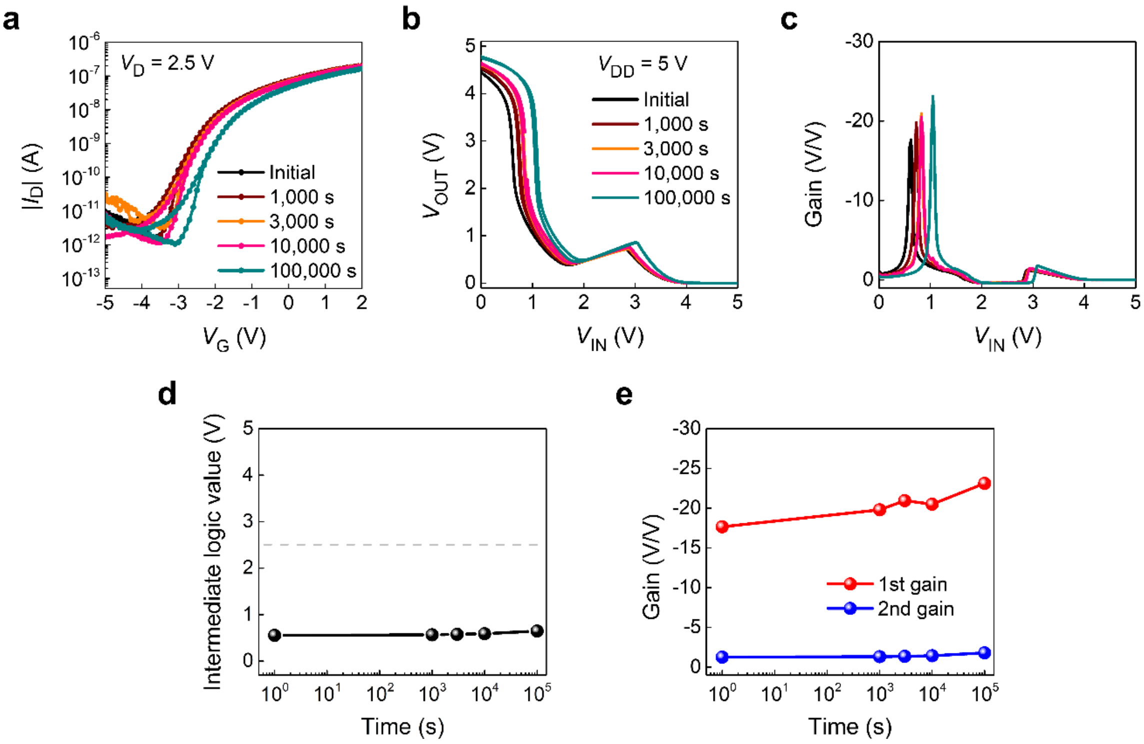

Supplementary Figure 22 | a. The transfer curves of the flash memory, **b**. VTCs and **c**. DC gain profile of the 3D T-inverter according to time. **d**. The intermediate logic value, **e**. 1st and 2nd gain values versus time extracted from VTCs.

The DC gain profiles of the flash memory in programming state over time

The changes in DC gain profiles of the flash memory with the optimum programming state (V_{prg}=+19 V) are shown in Supplementary Fig. **23**. Only negligible change was observed in the DC gain values (less than 4.5 and 3.0 V/V for the 1st and 2nd gain values, respectively) and their V_{IN} positions (less than 0.35 V) even after 10^5 s.

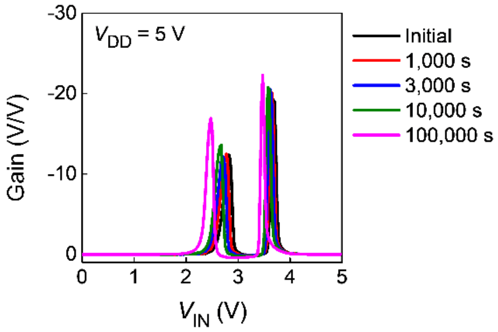

Supplementary Figure 23 | The DC gain profiles of the 3D T-inverter with the optimum memory programming state (V_{prg}=+19 V) according to time.

Comparison of the T-inverter performance

Supplementary Table **1** summarizes the performance of 3D T-inverter developed in this study compared to the previously reported high-performance non-Si-based T-inverters.

Supplementary Table 1 | Summary of the electrical characteristics of the reported non-Si-based T-inverters

Ref	V_{IN}/ V_{OUT} (V)	Output swing	V_{DD} (V)	Intermediate logic value (V)	DC gain (V/V)	Transient measurement	3D stacking
[8]	50 / 50	~100%	50	~23 (~V_{DD}/2)	>20	O	X
[9]	1 / 0.9	~90%	1	~0.5 (~V_{DD}/2)	4.5	X	X
[10]	5 / 5	~95%	5	~2.5 (~V_{DD}/2)	>1	O	X
[11]	4 / 8	~80%	10	~2	N/A*	X	X
[12]	25 / 1.7	~90%	2	~1 (~V_{DD}/2)	N/A	X	X
[13]	1 / 2	~90%	2	~1.1 (~V_{DD}/2)	12	X	X
[14]	60 / 20	~60%	26	22	1.2	X	X
[15]	40 / 2	100%	2	~0.9 (~V_{DD}/2)	0.2	X	X
[16]	80 / 8	100%	8	2	N/A	X	X
[17]	80 / 10	~60%	10	5 (~V_{DD}/2)	1	X	X
[18]	2 / 2	~95%	2	0.8, 1.2 (~V_{DD}/2)	N/A	O	X
**This work**	**5 / 5**	**100%**	**5**	**2.5 (~V_{DD}/2)**	**~20**	**O**	**O**

*DC gain value was not provided in the paper.

Summary of the switching characteristics of the T-inverter

The parameters for the switching speed of the T-inverter extracted from Supplementary Fig. **21** are shown in Supplementary Table **2**. A switching speed within a few hundreds of milliseconds was obtained, which is comparable to those obtained generally in the organic binary logic circuits.[19-21]

Supplementary Table 2 | Summary of the parameters for the switching speed of the T-inverter.

**Switching number**	**V_{OUT} (V)**		**Time (s)**		**Switching speed (ms)**
	**initial**	**terminal**	**initial**	**terminal**	
1	0	2.6	0.60	0.82	220
2	2.6	5	1.19	1.31	120
3	5	2.6	1.80	2.09	290
4	0	5	2.99	3.12	130
5	5	0	3.60	3.91	310
6	0	2.6	4.19	4.41	220
7	2.6	5	4.81	5.12	310

Comparison of the retention characteristics

Supplementary Table **3** summarizes the retention characteristics of the flash memory developed in this study compared to the previously reported organic flash memories employing polymer dielectric materials.

Supplementary Table 3 | Summary of the retention characteristics of the reported floating-gate flash memories based on polymer dielectrics.

Year[reference]	V_{prg}, V_{prg} (V)	$I_{10,000s}/I_{0s}$	$I_{100,000s}/I_{0s}$
2009[22]	30	0.544	N/A
2010[23]	90	0.448	0.316
2013[24]	70	0.291	N/A
	70	0.126	
2014[25]	6	0.697	0.612
2015[26]	50	0.552	N/A
	50	0.485	
	50	0.035	
2015[27]	50	0.428	N/A
2015[28]	80	0.637	0.386
2016[29]	150	0.175	N/A
	150	0.119	
	150	0.109	
	150	0.059	
2018[30]	60	0.149	N/A
2018[31]	80	0.303	N/A
	80	0.071	
	80	0.010	
2021[32]	30	0.599	N/A
	30	0.528	
**This work**	**19**	**0.911**	**0.716**

Supplementary References

1. Choi J. et al. Flexible, low-power thin-film transistors made of vapor-phase synthesized high-*k*, ultrathin polymer gate dielectrics. *ACS Appl. Mater. Interfaces* **9**, 20808-20817 (2010).
2. Moon H. et al. Synthesis of ultrathin polymer insulating layers by initiated chemical vapour deposition for low-power soft electronics. *Nat. Mater.* **14**, 628-635 (2015).
3. Lee S., Seong H., Im S. G., Moon H. & Yoo S. Organic flash memory on various flexible substrates for foldable and disposable electronics. *Nat. Commun.* **8**, 1-9 (2017).
4. Yang S. C., et al. Large-scale, low-power nonvolatile memory based on few-layer MoS_2 and ultrathin polymer dielectrics. *Adv. Electron. Mater.* **5**, 1800688 (2019).
5. Han H. & Kim C.-H. Unexpected Benefits of Contact Resistance in 3D Organic Complementary Inverters. *Adv. Electron. Mater.* **6**, 1900879 (2020).
6. Wang H. & Yu C. Organic Thermoelectrics: Materials Preparation, Performance Optimization, and Device Integration. *Joule* **3**, 53-80 (2019).
7. Wang Y., et al. Enhanced performance of organic field-effect transistor memory by hole-barrier modulation with an n-type organic buffer layer between pentacene and polymer electret. *Adv. Electron. Mater.* **6**, 1901184 (2019).
8. Yoo H., On S., Lee S. B., Cho K. & Kim J. J. Negative Transconductance Heterojunction Organic Transistors and their Application to Full-Swing Ternary Circuits. *Adv. Mater.* **31**, 1808265 (2019).
9. Nourbakhsh A., Zubair A., Dresselhaus M. S. & Palacios T. Transport properties of a MoS2/WSe2 heterojunction transistor and its potential for application. *Nano Lett.* **16**, 1359-1366 (2016).
10. Lee L. et al. ZnO composite nanolayer with mobility edge quantization for multi-value logic transistors. *Nat. Commun.* **10**, 1998 (2019).
11. Kobashi K., Hayakawa R., Chikyow T. & Wakayama Y. Multi-valued logic circuits based on organic anti-ambipolar transistors. *Nano Lett.* **18**, 4355-4359 (2018).
12. Shim J. et al. Phosphorene/rhenium disulfide heterojunction-based negative differential resistance device for multi-valued logic. *Nat. Commun.* **7**, 13413 (2016).

13. Huang M. et al. Multifunctional high-performance van der waals heterostructures. *Nat. Nanotechnol.* **12**, 1148-1154 (2017).

14. Duong N. T. et al. Modulating the functions of $MoS_2/MoTe_2$ van der waals heterostructure via thickness variation. *ACS Nano* **13**, 4478-4485 (2019).

15. Wu E. et al. Photoinduced doping to enable tunable and high-performance anti-ambipolar $MoTe_2/MoS_2$ heterotransistors. *ACS Nano* **13**, 5430-5438 (2019).

16. Kim J. Y. et al. Distinctive field-effect transistors and ternary inverters using cross-type WSe_2/MoS_2 heterojunctions treated with polymer acid. *ACS Appl. Mater. & Interfaces* **12**, 36530-36539 (2020).

17. Park C.-J. et al. Photo-responsive MoS_2/organic-rubrene heterojunction field-effect-transistor: Application to photo-triggered ternary inverter. *Semicond. Sci. Technol.* **35**, 065020 (2020).

18. Kim B. Inkjet-printed ternary inverter circuits with tunable middle logic voltages. *Adv. Electron. Mater.* **6**, 2000426 (2020).

19. Zschieschang U., Bader V. P. & Klauk H. Below-one-volt organic thin-film transistors with large on/off current ratios. *Org. Electron.* **49**, 179-186 (2017).

20. Chang J. S., Facchetti A. F. & Reuss R. A circuits and systems perspective of organic/printed electronics: review, challenges, and contemporary and emerging design approaches. *IEEE J. Emerg. Sel. Top.* **7**, 7-26 (2017).

21. Kumar B., Kaushik B. K. & Negi Y. S. Organic thin film transistors: structures, models, materials, fabrication, and applications: a review. *Polym. Rev.* **54**, 33-111 (2014).

22. Mabrook M. F., Yun Y., Pearson C., Zeze D. A. & Petty M. C. A pentacene-based organic thin film memory transistor. *Appl. Phys. Lett.* **94**, 122 (2009).

23. Kim S.-J. & Lee J.-S. Flexible organic transistor memory devices. *Nano Lett.* **10**, 2884-2890 (2010).

24. Kang M., Baeg K. J., Khim D., Noh Y. Y. & Kim D. Y. Printed, flexible, organic nano-floating-gate memory: Effects of metal nanoparticles and blocking dielectrics on memory characteristics. *Adv. Funct. Mater.* **23**, 3503-3512 (2013).

25. Li J. & Yan F. Solution-processable low-voltage and flexible floating-gate memories based on an n-type polymer semiconductor and high-*k* polymer gate dielectrics. *ACS Appl. Mater. & Interfaces* **6**, 12815-12820 (2014).

26. Shih C. C., Chiu Y. C., Lee W. Y., Chen J. Y. & Chen W. C. Conjugated polymer nanoparticles as nano floating gate electrets for high performance nonvolatile organic transistor memory devices. *Adv. Funct. Mater.* **25**, 1511-1519 (2015).

27. Kang M. et al. Synergistic high charge-storage capacity for multi-level flexible organic flash memory. *Sci. Rep.* **5**, 12299 (2015).

28. Park Y., Park S., Jo I., Hong B. H. & Hong Y. Controlled growth of a graphene charge-floating gate for organic non-volatile memory transistors. *Org. Electron.* **27**, 227-231 (2015).

29. Yi M. et al. The effect of porous structure of PMMA tunneling dielectric layer on the performance of nonvolatile floating-gate organic field-effect transistor memory devices. *Org. Electron.* **33**, 95-101 (2016).

30. Wang G., Liu X. & Wang W. Solution processed organic transistor nonvolatile memory with a floating-gate of carbon nanotubes. *IEEE Electron Device Lett.* **39**, 111-114 (2017).

31. Wang K. et al. A centimeter-scale inorganic nanoparticle superlattice monolayer with non-close-packing and its high performance in memory devices. *Adv. Mater.* **30**, 1800595 (2018).

32. Kwon H. j. et al. Newly synthesized nonvacuum processed high-*k* polymeric dielectrics with carboxyl functionality for highly stable operating printed transistor applications. *Adv. Funct. Mater.* **31**, 2007304 (2021).
